# Factors Affecting Health-Promoting Behaviors among Nursing Students

**DOI:** 10.3390/ijerph17176291

**Published:** 2020-08-28

**Authors:** Younghui Hwang, Jihyun Oh

**Affiliations:** 1 Department of Nursing, University of Ulsan, Ulsan 44610, Korea; hyh77@ulsan.ac.kr; 2 Department of Nursing, Daejeon University, Daejeon 300-716, Korea

**Keywords:** health-promoting behaviors, health perceptions, health concern, nursing education, nursing students

## Abstract

Health-promoting behaviors help prevent chronic illness. Health-promoting behaviors of nursing students can affect not only their own health, but also the health of their future patients, for whom they can act as role models. Nursing students should participate in health-promoting behaviors; however, nursing students often have unhealthy behaviors. This study aimed to investigate the factors affecting health-promoting behaviors in nursing students. A descriptive, self-report survey of 304 nursing students from three universities in South Korea was conducted. Subjects’ general characteristics, health perceptions, health concerns, and health-promoting behaviors were collected. Of the total participants, 90.1% were female and the mean age was 20.4 years. The mean score for health-promoting behaviors was 2.47, higher than the midpoint. The mean for the subscale of physical activity among health-promoting behaviors was the lowest. The main factors affecting health-promoting behaviors were gender, health perceptions, health concern, and time per week spent searching online for health-related information. The main factors affecting physical activity were gender, health concern, and time per week spent searching online for health-related information. Based on the study findings, it is recommended that a program to empower nursing students to perform health-promoting behaviors be incorporated into the nursing education curriculum with regard to unique needs based on gender. Specifically, it would be effective to develop programs that are easily accessible via the Internet.

## 1. Introduction

A health-promoting lifestyle refers to self-directed behaviors that can help prevent chronic illness and lead to practicing health-promoting behaviors [1]. Health-promoting behaviors consist of six dimensions of health: responsibility, physical activity, nutrition, interpersonal relations, spiritual growth, and stress management [2]. Health-promoting behaviors is a significant concept required in nursing [3]. Nursing students not only have responsibilities as role models to provide clients with health promotion advice, but also should understand the significance of their own health-promoting behaviors as role models of public health [4,5]. 

Nursing students continuously learn about health promotion in the nursing curriculum. In nursing education, nursing students’ understanding and knowledge about health promotion are expected to deepen according to the development of a nursing curriculum [4]. Both nurses and pre-registered nurses have unhealthy lifestyles including poor nutrition, smoking, alcohol consumption, and physical inactivity [6,7]. Nursing students have the same unhealthy behaviors [8,9,10,11]. If interventions to encourage healthy behaviors among the nurses of the future are developed and implemented, it can be the foundation for maintaining health-promoting behaviors when they become nurses. As future healthcare professionals, nursing students play an important role in public health for others, in addition to their own health [8]. Therefore, this study focused attention on the health-promoting behaviors of nursing students. 

Nurses are well informed about the importance of health-promoting behaviors [12], and it is thought that nursing students also have sufficient knowledge about the importance of these behaviors. However, it is not easy for knowledge to lead to action [12]. Therefore, many studies are being conducted to analyze the factors affecting the health promotion behaviors of nursing students in order to enhance their health promotion efforts [13]. Variables affecting health promotion behaviors included marital status [4]; perceived disability, perceived self-efficacy, perceived social support, clinical practice stress [13]; and family function and health perceptions [14]. However, no strategies have been identified that can be used to effectively improve the health-promoting behaviors of nursing students.

*Health perceptions* refer to a person’s overall assessment of her or his own health [15]. It is used as an indicator of self-assessed health status because subjective perceptions of one’s own health may reveal actual current health status [16]. The relationship between perceived current health status and health-promoting behaviors has been reported in various studies. Individuals with better health status were more likely to perform positive health behaviors [17,18,19], but others reported that they did not [12]. 

*Health concern* refers to the level of personal interest in health [20]. Studies on the relationship between health concern and health-promoting behaviors in adults have shown that a higher level of health concern results in better perceptions of one’s own health status and a higher level of health-promoting behaviors [17,21]. Health concern is thought to be a very important factor that influences health-promoting behaviors.

It can be inferred based on this prior research that health perceptions, and health concern may be associated with health-promoting behaviors in nursing students. However, despite the increasing number of studies on health-promoting behaviors of nursing students, studies have not been sufficiently conducted to date to identify the relationship between health perceptions, health concern, and health-promoting behaviors among nursing students. Hence, the current study aimed to investigate the relationships among health perceptions, health concern, and health-promoting behaviors in nursing students and to investigate their effects on health-promoting behaviors.

## 2. Materials and Methods

### 2.1. Study Design and Sample

This study used a cross-sectional, self-report survey to investigate the relationships among health perceptions, health concern, and health-promoting behaviors in nursing students, and assessing the effects of different variables on health-promoting behaviors.

The study sample was a convenience sample of nursing students recruited from three universities in South Korea. The required sample size was estimated using G power 3.1 (Heinrich-Heine-Universität Düsseldorf, Düsseldorf, Germany) [22]. The minimum required sample size for multiple regression analysis was 138 under the assumptions of a significance level of 0.05, an effect size of 0.15, and a power of 0.95. In consideration of an anticipated dropout rate of 10% and incomplete responses, the survey was distributed to 320 nursing students. Data from a total of 304 subjects were submitted for final analysis after 16 nursing students were excluded due to missing data and incomplete responses.

### 2.2. Measures

#### 2.2.1. Health-Promoting Behaviors

The Health-Promoting Lifestyle Profile (HPLP-Ⅱ), developed by Walker, Sechrist, and Pender [23] and translated to Korean by Seo [24], was used to assess health-promoting behaviors. The instrument consists of a total of 52 items across six subdomains (health responsibility—nine items; physical activity—eight items; nutrition—nine items; for spiritual growth—nine items; interpersonal relations—nine items; and stress management—eight items). Each item was evaluated on a four-point Likert scale, with “never” assigned 1 point, “sometimes” assigned 2 points, “often” assigned 3 points, and “routinely” assigned 4 points. Higher mean scores indicate higher engagement in health-promoting behaviors. Cronbach’s α was 0.92 in Seo’s study [24] and 0.94 in the current study, thus demonstrating high reliability.

#### 2.2.2. Health Perceptions

*Health perceptions* refer to personal meanings that govern an individual’s health behavior [25]. In this current study, health perceptions were measured using the scale for current health revised by Oh and Yi [26], which is a subscale of the Health Perceptions Questionnaire originally developed by Ware [25]. One specific item selected from the scale regarding self-assessment of current health status, “I am confident with my health”, was used. The item was evaluated on the four-point Likert-type scale, ranging from 1 = “not at all confident” to 4 = “very confident”, and the higher the score, the more positive the perceived health.

#### 2.2.3. Health Concern

Level of perceived health concern was assessed with one item: “How interested are you in perceiving yourself to be in good health?” The item was measured on a 4-point scale, with 1 point for “not interested” and 4 points for “highly interested”. The higher the score, the higher the interest in health.

#### 2.2.4. Data Collection

Data were collected from 10 June through 25 June 2020. The researcher informed a total of 320 nursing students recruited from three universities in South Korea about the necessity of obtaining voluntary consent for participation in the study, the study purpose, and the estimated time needed to complete the survey; thereafter, the survey was conducted directly with those who consented to participate. Completing the survey took 10–15 min; 16 of the 320 returned surveys were discarded owing to incomplete responses, resulting in a total of 304 surveys (95% response rate) submitted for final analysis. We noted that 16 of those who did not complete or return the survey did not accurately reflect the demographics.

#### 2.2.5. Data Analysis

Data were analyzed using IBM SPSS Statistics software Version 23.0 (IBM Corp., Armonk, NY, USA). Subjects’ general characteristics, health perceptions, health concern, and health-promoting behaviors were examined by descriptive statistics. Differences in health-promoting behaviors according to subjects’ general characteristics were examined using the independent t-test and analysis of variance (ANOVA) with a statistically significant level of 5%, and the Kolmogorov–Smirnov test was used to examine the normality of data. The correlations between health perceptions, health concern, and health-promoting behaviors were examined by computing Pearson’s correlation coefficients. Stepwise multiple regression was performed to identify factors influencing health-promoting behaviors.

#### 2.2.6. Ethical Considerations

The study was approved by the Daejeon University Institutional Review Board (1040647-202004-HR-005-03) and complied with the ethical guidelines delineated in the Declaration of Helsinki. Subjects were informed of the details about study purposes and procedures and signed a written consent form before the survey was administered. Subjects were also informed about the benefits and risks of participation and could withdraw from the study at any time; moreover, they were informed that the data would be used only for research purposes and that anonymity and autonomy would be guaranteed. Surveys were distributed only to those who consented to study participation. Returned surveys were stored in a secure place. 

## 3. Results

### 3.1. Subjects’ General Characteristics and Differences

Regarding gender, 90.1% were female. The age range was 17–40 years, with a mean age of 20.4 ± 2.3 years. Regarding academic standing, 25.3% were freshmen, 20.7% sophomores, 27% juniors, and 27% seniors. Majority of subjects (60.5%) was unaffiliated with any religious group. Regarding daily internet usage hours, the most common response was over two hours but fewer than three hours (29.9%), and most subjects (60.2%) responded that they spent less than one hour per week searching online for health-related information (Table 1). 

Regarding differences in health-promoting behaviors according to subjects’ general characteristics, there was a significant difference depending on gender (t = 3.09, *p* < 0.01) and time per week spent searching online for health-related information (F = 7.90, *p* < 0.01). Post hoc testing showed that subjects spending less than one hour per week for this purpose had a mean score of 2.38 ± 0.42, significantly lower in comparison to subjects spending one hour or more but fewer than three hours and to those spending three hours or more but fewer than six hours. 

In the subdomains of health-promoting behaviors, regarding differences in health responsibility according to nursing students’ general characteristics, there was a significant difference depending on gender (t = 2.81, *p* < 0.01), grade level (F = 4.37, *p* < 0.01), and time per week spent searching online for health-related information (F = 2.75, *p* = 0.04). Post hoc testing showed that health responsibility was higher in seniors, 2.34 ± 0.63, compared with freshmen 2.02 ± 0.47. Regarding differences in physical activity according to general characteristics, gender (t = 5.97, *p* < 0.01) and time per week spent searching online for health-related information (F = 42.17, *p* < 0.01) were statistically significant in health-promoting behaviors according to general characteristics. Post hoc test results revealed that subjects spending less than one hour per week had a mean score of 1.80 ± 0.58, a level statistically significantly lower in comparison to subjects spending one hour or more but fewer than three hours and with a mean score of 2.42 ± 0.69, and those spending three hours or more but fewer than six hours, and those spending more than six hours. Additionally, nutrition showed a statistically significant difference according to gender (t = 2.58, *p* = 0.01), and time per week spent searching online for health-related information (F = 6.07, *p* < 0.01). Post hoc testing showed that subjects spending less than one hour per week had a mean score of 2.05 ± 0.60, a level statistically significantly lower in comparison to subjects spending one hour or more but fewer than three hours and with a mean score of 2.35 ± 0.60 (Table 2). 

### 3.2. Levels of Health Perceptions, Health Concern, and Health-Promoting Behaviors

The mean score for health perceptions was 2.61 ± 0.76 and the mean score for health concern was 2.59 ± 0.76. The overall mean score for health-promoting behavior was 2.47 ± 0.44. Among six dimensions of health-promoting behaviors, high scores were reported for interpersonal support and lower scores for physical activity (Table 3).

### 3.3. Correlations between Health Perceptions, Health Concern, and Health-Promoting Behaviors

Health perceptions showed statistically significant positive correlations with health concern (*r* = 0.19, *p* < 0.01), and health-promoting behaviors (*r* = 0.24, *p* < 0.01). Health concern showed statistically significant positive correlations with health-promoting behaviors (*r* = 0.35, *p* < 0.01).

### 3.4. Factors Influencing Health-Promoting Behaviors

The outcomes of the stepwise regression analysis were as follows. 

The most powerful factor influencing health-promoting behaviors was health concern (β = 0.29, *p* < 0.01). The next most powerful factor was health perceptions (β = 0.16, *p* < 0.01), followed by and time per week spent searching online for health-related information (β = 0.16, *p* < 0.01), and, finally, male gender (β = −0.12, *p* = 0.03). The explanatory power of these variables was 19.2% (F = 18.97, *p* < 0.01, R^2^ = 0.202, Adj. R^2^ = 0.192), as shown in Table 4. 

In the subdomains of health-promoting behaviors, the factors influencing health responsibility were male gender (β = −0.29, *p* < 0.01), grade level (β = 0.10, *p* < 0.01), and health concern (β = 0.22, *p* < 0.01). The explanatory power of these variables was 13.7% (F = 17.5, *p* < 0.01, R^2^ = 0.146, Adj. R^2^ = 0.137). The factors influencing physical activity were male gender (β = −0.53, *p* < 0.01), time per week spent searching online for health-related information (β = 0.48, *p* < 0.01), and health concern (β = 0.21, *p* < 0.01). The explanatory power of these variables was 36.8% (F = 59.91, *p* < 0.01, R^2^ = 0.375, Adj. R^2^ = 0.368). The factors influencing nutrition were time per week spent searching online for health-related information (β = 0.15, *p* < 0.01), and health concern (β = 0.22, *p* < 0.01). The explanatory power of these variables was 10.9% (F = 19.44, *p* < 0.01, R^2^ = 0.114, Adj. R^2^ = 0.109). The factor influencing spiritual growth was health perceptions (β = 0.16, *p* < 0.01). The explanatory power of these variables was 9.9% (F = 17.68, *p* < 0.01, R^2^ = 0.105, Adj. R^2^ = 0.099). The factor influencing interpersonal support was health perceptions (β = 0.13, *p* < 0.01). The explanatory power of these variables was 7.0% (F = 12.36, *p* < 0.01, R^2^ = 0.076, Adj. R^2^ = 0.070). The factor influencing stress management was health perceptions (β = 0.13, *p* < 0.01). The explanatory power of these variables was 7.0% (F = 12.44, *p* < 0.01, R^2^ = 0.076, Adj. R^2^ = 0.070) (Table 4).

## 4. Discussion

Nursing students should participate in health-promoting behaviors for the benefit of their own health as future healthcare professionals. This study evaluated health perceptions, health concern, and health-promoting behaviors of nursing students in Korea, and the factors impacting health-promoting behaviors.

In this study, the mean for health-promoting behaviors of nursing students was 2.47 (0.44) out of 4 points, a level higher than the midpoint. This finding was similar to previous study findings [4,27,28]. The mean score for health-promoting behaviors of nursing students was similar to that of general university students in South Korea [29]. Among the subscale of health-promoting behaviors, the mean score of interpersonal support was highest, and the mean scores of health responsibility and physical activity were the lowest, similar to findings in general university students in South Korea [29]. There was no difference in health-promoting behaviors according to grade level, which is similar to the findings of Hosseini et al. [4]. This finding suggested that, despite the nursing students’ knowledge of the importance of health promotion, this knowledge does not always translate to self-care. A previous study also found that the knowledge of health-promoting behaviors of nursing students did not lead to their own health-promoting behaviors [29,30]. It is not easy to participate in health-promoting behaviors as personal and environmental factors, including past experience, fatigue, and institutional support or norms, can interfere with health promotion behaviors [12]. However, if nursing students can recognize the factors that hinder their health-promoting behaviors and participate in health-promoting behaviors by controlling them, it can be of great help to encourage patients to perform health-promoting behaviors in the future. Currently, nursing education focuses on knowledge related to health-promoting behaviors and education regarding skills [30]. To participate in health-promoting behaviors, empowerment to help overcome obstacles in personal and environment factors and make decisions to maintain a healthy life is necessary [12,31]. Therefore, further research is needed to explore the personal and environmental factors that influence nursing students’ health-promoting behaviors. Additionally, it will be necessary for nursing education to combine knowledge of health-promoting behaviors with empowerment for participating in health-promoting behaviors.

This study found that the mean of the subscale for physical activity among health-promoting behaviors was the lowest, and the mean of the subscale for interpersonal support was the highest. This finding is similar to that of Hosseini et al. [4] and Hong [29]. This finding suggests that South Korea also lacks good policies for leisure time and attention by individuals and authorities to the importance of physical activity as reported by Hosseini et al. [4]. Since physical activity is the most unfulfilled health-promoting behavior [4,29,30], it is thought that attention should be paid to interventions that enhance physical activity.

This study showed that the main factors affecting health-promoting behaviors were gender, health conception, health concern, and time per week spent searching online for health-related information. In the subdomains of health-promoting behaviors, the main factors affecting physical activity that was not perform well were gender, health concern, and time per week spent searching online for health-related information. In this study, men scored higher than women in physical activity. This finding is similar to Hosseini et al. [4] and Hong [29]. The level of physical activity for adult women is lower than that of adult men in almost all countries [32]. This finding indicates that efforts to resolve gender inequalities in physical activity have continued [33], but the problem has not yet been resolved. Since the proportion of female nursing students is relatively higher than that of male students in Korea, it is thought that a gender-based approach is needed when developing policies or programs to increase physical activity in nursing students.

Time per week spent searching online for health-related information also impacted physical activity. This study was conducted with undergraduate students, who most actively use information found via the Internet, such as social media, blogs, Internet cafes, and online communities. This study has significance in that the subjects showed a higher level of physical activity by understanding and using health information via the Internet. It is expected that the Internet may go beyond simply providing information online and play a role in public health education given the reality of the present information age of web searches for health information without temporal and spatial restrictions and with easy acquisition of information [34]. Therefore, in the future, the Internet could be used as a platform to disseminate strategies for improving health-promoting behaviors for nursing students.

The main factor affecting spiritual growth, which is the second poorest performer among subdomains of health-promoting behaviors, was health perceptions. Since health perceptions can be the motivation for health-promoting behaviors, it may be assumed that a high level of health perceptions will increase nursing student willingness to carry out health-promoting behaviors [35]. In particular, this study found that health perceptions affect not only spiritual growth, but also interpersonal support and stress management. However, nurse health perceptions typically are not high, and their actual health conditions are not good [36]. High health perceptions provide an incentive to conduct health-promoting behaviors, so it is thought that nursing students need support to maintain their health.

Although the study found no grade level differences in health-promoting behaviors, there were grade level differences in health responsibility. The results demonstrated that grade level affects health responsibility. In this study, health responsibility was higher in seniors than in freshmen. This finding supported those of a previous study [4]. It can be assumed that nursing students increased their health responsibilities as they were educated by the nursing curriculum on the importance of health promotion. In other words, nursing students have a sense of responsibility for their own health, but this is still insufficient to support participation in health-promoting behaviors. Therefore, it is believed that an educational program that can increase the health-promoting behaviors of nursing students should be incorporated into the nursing education curriculum.

This study found that the higher health perceptions and the higher health concern, the better the health-promoting behaviors. This finding is consistent with previous studies [17,37]. In particular, health concern is a major variable influencing physical activity, so it is thought that a program or institutional support is needed to support nursing students to be steadfastly interested in their health and to promote health-promoting behaviors. On the other hand, studies are being conducted to increase health-promoting behaviors for medical students, future doctors who can be role models for their patients, like nursing students [38,39]. A comprehensive health-promoting program for health and wellbeing has been developed and implemented in line with the curriculum of medical students [40]. However, few institutional programs have been developed and applied to improve health-promoting behaviors among nursing students. Therefore, it is necessary to consider developing and implementing programs that can increase health-promoting behaviors of nursing students in the curriculum.

Finally, education using the Internet is thought to be helpful to motivate health-promoting behaviors of nursing students. Beyond simply providing health information, specific programs will need to be developed to foster physical activity and to empower nursing students to maintain their health; simultaneously, public health programs should be customized based on the analysis of both sexes to facilitate easy use by nursing students of social media applications in articulating and communicating their health concern. Such programs may increase interest in health, proper searching, and information use.

### Limitations and Recommendations

The study has a few limitations. First, the sample was a convenience sample of nursing students from three universities, and the proportions of male and female students were unbalanced. Generalization of the study finding is difficult because the small and convenient sample might not represent the entire population of nursing students in South Korea. Accordingly, a future study should balance the proportions of male and female students to examine the factors affecting health-promoting behaviors. Second, health perceptions and health concern were defined as subjectively perceived competencies, and a self-reporting method was used to collect the relevant data. Hence, the data may not reflect the actual knowledge level with respect to obtaining and using health information and the actual health status. Nevertheless, the study is of significance in that self-reported health perceptions, health concern, and health-promoting behaviors in nursing students were examined; their relationships were investigated; and the data acquired were useful in exploring effective ways to enhance health-promoting behaviors.

## 5. Conclusions

The current study was conducted to examine the factors affecting health-promoting behaviors in nursing students. In this study, the mean score for health-promoting behaviors was a level higher than the midpoint, and the mean of physical activity was lowest among the subdomains of health-promoting behaviors. Therefore, attention is needed to develop programs to improve physical activity. The main factor affecting health-promoting behaviors were gender, health conception, health concern, and time per week to spend searching for health-related information. The main factors affecting physical activity were gender, health concern, and time per week spent searching for health-related information. Based on the study findings, it is suggested that a program that could empower nursing students to perform health-promoting behaviors in the nursing education curriculum should be developed in consideration of gender. Specifically, it would be effective to develop internet programs that can induce health concern and provide strategies for improving health-promoting behaviors.

## Figures and Tables

**Table 1:** Subjects’ general characteristics (N = 304).

Variable	Statistics	Category	Total Sample
Age (years)	Mean (SD)	Range: 17–40	20.4 (2.3)
Gender	N (%)	Male	30 (9.9)
		Female	274 (90.1)
Grade level	N (%)	Freshmen	77 (25.3)
		Sophomore	63 (20.7)
		Junior	82 (27.0)
		Senior	82 (27.0)
Religion	N (%)	Yes	120 (39.5)
		No	184 (60.5)
Daily Internet usage hours	N (%)	<1	18 (5.9)
		1–2	79 (26.0)
		2–3	91 (29.9)
		3–4	66 (21.7)
		≥4	50 (16.4)
Time per week spent searching online for health-related information	N (%)	<1	183 (60.2)
		1–3	97 (31.9)
		3–6	19 (6.3)
		≥6	5 (1.6)

**Table 2:** Differences in health-promoting behaviors according to general characteristics (N = 304).

		Health-Promoting Behaviors						
		Total	HR ^1^	PA ^2^	Nu ^3^	SG ^4^	IS ^5^	SM ^6^
Variable	Category	Mean (SD)						
Gender	Male	2.71 (0.57)	2.47 (0.66)	2.83 (0.83)	2.45 (0.77)	2.97 (0.61)	3.19 (0.61)	2.70 (0.68)
	Female	2.44 (0.43)	2.16 (0.57)	2.02 (0.69)	2.14 (0.60)	2.89 (0.63)	3.17 (0.56)	2.55 (0.58)
	*df*	302	302	302	302	302	302	302
	t(*p*)	3.09 (<0.01)	2.81 (<0.01)	5.97 (<0.01)	2.58 (0.01)	0.60 (0.55)	0.17 (0.87)	1.32 (0.19)
Grade level	Freshmen	2.44 (0.35)	2.02 (0.47)	2.03 (0.62)	2.16 (0.56)	2.91 (0.49)	3.25 (0.48)	2.54 (0.52)
	Sophomore	2.42 (0.50)	2.13 (0.62)	1.99 (0.73)	2.10 (0.65)	2.91 (0.68)	3.14 (0.64)	2.54 (0.64)
	Junior	2.47 (0.47)	2.24 (0.58)	2.17 (0.78)	2.13 (0.62)	2.88 (0.67)	3.16 (0.57)	2.55 (0.63)
	Senior	2.53 (0.45)	2.34 (0.63)	2.10 (0.81)	2.28 (0.67)	2.90 (0.67)	3.13 (0.56)	2.63 (0.58)
	*df*	303	303	303	303	303	303	303
	F(*p*)	0.75(0.52)	4.37 (<0.01)a<b	1.17 (0.32)	1.22 (0.30)	0.33 (0.99)	0.74 (0.53)	1.12 (0.35)
Religion	Yes	2.49 (0.48)	2.24 (0.64)	2.13 (0.81)	2.22 (0.67)	2.93 (0.64)	3.15 (0.61)	2.56 (0.60)
	No	2.45 (0.42)	2.15 (0.55)	2.07 (0.70)	2.14 (0.60)	2.88 (0.62)	3.18 (0.53)	2.57 (0.58)
	*df*	302	302	302	302	302	302	302
	t(*p*)	0.75 (0.46)	1.22(0.22)	0.66 (0.51)	1.02 (0.31)	0.60 (0.55)	−0.47 (0.64)	−0.15 (0.88)
Daily Internet usage hours	<1	2.51 (0.38)	2.27 (0.48)	2.11 (0.61)	2.25 (0.50)	2.92 (0.74)	3.20 (0.64)	2.55 (0.64)
	1–2	2.56 (0.48)	2.28 (0.65)	2.24 (0.81)	2.32 (0.68)	2.99 (0.59)	3.18 (0.58)	2.67 (0.63)
	2–3	2.43 (0.43)	2.10 (0.57)	2.04 (0.71)	2.15 (0.60)	2.88 (0.67)	3.13 (0.57)	2.54 (0.58)
	3–4	2.46 (0.40)	2.20 (0.58)	2.10 (0.75)	2.06 (0.59)	2.93 (0.54)	3.21 (0.51)	2.55 (0.55)
	≥4	2.39 (0.47)	2.17 (0.57)	1.96 (0.70)	2.10 (0.65))	2.76 (0.67)	3.15 (0.58)	2.50 (0.58)
	*df*	303	303	303	303	303	303	303
	F(*p*)	1.49 (0.21)	1.16 (0.33)	1.22 (0.30)	1.94 (0.10)	1.12 (0.35)	0.22 (0.93)	0.81 (0.52)
Time per week spent searching online for health-related information	<1	2.38 (0.42)	2.11 (0.58)	1.80 (0.58)	2.05 (0.60)	2.85 (0.67)	3.16 (0.56)	2.53 (0.62)
	1–3	2.58 (0.43)	2.30 (0.58)	2.42 (0.69)	2.35 (0.60)	2.97 (0.54)	3.16 (0.58)	2.60 (0.51)
	3–6	2.70 (0.45)	2.36 (0.66)	3.03 (0.62)	2.35 (0.65)	2.99 (0.13)	3.28 (0.49)	2.62 (0.65)
	≥6	2.81 (0.64)	2.20 (0.37)	3.30 (0.68)	2.53 (0.99)	2.95 (0.79)	3.45 (0.56)	2.87 (0.82)
	*df*	303	303	303	303	303	303	303
	F(*p*)	7.90 (<0.01) c < d,e	2.75 (0.04)	42.17 (<0.01)c < d < e,f	6.07 (<0.01)c < d	0.84 (0.47)	0.70 (0.56)	0.79 (0.50)

^1^ HR = health responsibility; ^2^ PA = physical activity; ^3^ Nu = nutrition; ^4^ SG = spiritual growth; ^5^ IS = interpersonal support; ^6^ SM = stress management; a = freshmen; b = senior; c = less than one hour per week; d = one hour or more but fewer than three hours per week; e = three hours or more but fewer than six hours per week; f = more than six hours per week.

**Table 3:** Levels of health perceptions, health concern, and health-promoting behaviors (N = 304).

Variables	Range	Mean (SD)
Health perceptions	1–4	2.61(0.76)
Health concern	1–4	2.59(0.76)
Health-promoting behavior	1.1–3.9	2.47(0.44)
Health responsibility	1–4	2.19(0.59)
Physical activity	1–4	2.10(0.74)
Nutrition	1–4	2.90(0.63)
Spiritual growth	1–4	2.17(0.63)
Interpersonal support	1–4	3.17(0.56)
Stress management	1–4	2.57(0.59)

**Table 4:** Factors affecting health-promoting behaviors (N = 304).

Variable	Total	HR ^1^	PA ^2^	Nu ^3^	SG ^4^	IS ^5^	SM ^6^
	β (SE)						
Constant	1.98 (0.20)	1.91 (0.25) **	1.85 (0.27)	1.39 (0.13) **	2.00 (0.16) **	2.48 (0.14) **	1.84 (0.15) **
Gender	−0.12 (0.08) *	−0.29 (0.11) **	−0.53 (0.12) **				
Grade level		0.10 (0.03) **					
Time per week spent searching online for health-related information	0.16 (0.04) **		0.48 (0.05) **	0.15 (0.05) **			
Health concern	0.29 (0.03) **	0.22 (0.04) **	0.21 (0.05) **	0.22 (0.05) **			
Health perceptions	0.16 (0.03) **				0.16 (0.05) **	0.13 (0.04) **	0.13 (0.04) **
R^2^	0.202	0.146	0.375	0.114	0.105	0.076	0.076
Adj. R^2^	0.192	0.137	0.368	0.109	0.099	0.070	0.070
F	18.97 **	17.05 **	59.91 **	19.44 **	17.68 **	12.36 **	12.44 **

^1^ HR=health responsibility; ^2^ PA=physical activity; ^3^ Nu=nutrition; ^4^ SG=spiritual growth; ^5^ IS=interpersonal support; ^6^ SM=stress management; * *p* < 0.05, ** *p* < 0.01.
